# Vineland adaptive behavior scales to identify neurodevelopmental problems in children with Congenital Hyperinsulinism (CHI)

**DOI:** 10.1186/s13023-017-0648-7

**Published:** 2017-05-22

**Authors:** Maria Salomon-Estebanez, Zainab Mohamed, Maria Michaelidou, Hannah Collins, Lindsey Rigby, Mars Skae, Raja Padidela, Stewart Rust, Mark Dunne, Karen Cosgrove, Indraneel Banerjee, Jacqueline Nicholson

**Affiliations:** 1 Department of Paediatric Endocrinology, Royal Manchester Children’s Hospital, Central Manchester University Hospitals, Oxford Road, Manchester, M13 9WL UK; 2 Department of Paediatric Endocrinology and Diabetes, Nottingham Children’s Hospital, Nottingham University Hospitals, Derby Road, Nottingham, NG7 2UH UK; 3 Paediatric Psychosocial Department, Royal Manchester Children’s Hospital, Central Manchester University Hospitals, Oxford Road, Manchester, M13 9WL UK; 4 Faculty of Biology, Medicine and Health, University of Manchester, Oxford Road, Manchester, M13 9PL UK

**Keywords:** Glucose, Insulin, Vineland, Development, Cognitive assessment, Neurodevelopment, Developmental delay

## Abstract

**Background:**

Congenital Hyperinsulinism (CHI) is a disease of severe hypoglycaemia caused by excess insulin secretion and associated with adverse neurodevelopment in a third of children. The Vineland Adaptive Behavior Scales Second Edition (VABS-II) is a parent report measure of adaptive functioning that could be used as a developmental screening tool in patients with CHI. We have investigated the performance of VABS-II as a screening tool to identify developmental delay in a relatively large cohort of children with CHI. VABS-II questionnaires testing communication, daily living skills, social skills, motor skills and behaviour domains were completed by parents of 64 children with CHI, presenting both in the early neonatal period (Early-CHI, *n* = 48) and later in infancy (Late-CHI, *n* = 16). Individual and adaptive composite (Total) domain scores were converted to standard deviation scores (SDS). VABS-II scores were tested for correlation with objective developmental assessment reported separately by developmental paediatricians, clinical and educational psychologists. VABS-II scores were also investigated for correlation with the timing of hypoglycaemia, gender and phenotype of CHI.

**Results:**

Median (range) total VABS-II SDS was low in CHI [-0.48 (-3.60, 4.00)] with scores < -2.0 SDS in 9 (12%) children. VABS-II Total scores correctly identified developmental delay diagnosed by objective assessment in the majority [odds ratio (OR) (95% confidence intervals, CI) 0.52 (0.38, 0.73), *p* < 0.001] with 95% specificity [area under curve (CI) 0.80 (0.68, 0.90), *p* < 0.001] for cut-off < -2.0 SDS, although with low sensitivity (26%). VABS-II Total scores were inversely correlated (adjusted R^2^ = 0.19, *p* = 0.001) with age at presentation (*p* = 0.024) and male gender (*p* = 0.036), males having lower scores than females in those with Late-CHI [-1.40 (-3.60, 0.87) v 0.20 (-1.07, 1.27), *p* = 0.014]. The presence of a genetic mutation representing severe CHI also predicted lower scores (R^2^ = 0.19, *p* = 0.039).

**Conclusions:**

The parent report VABS-II is a reliable and specific tool to identify developmental delay in CHI patients. Male gender, later age at presentation and severity of disease are independent risk factors for lower VABS-II scores.

## Background

Congenital Hyperinsulinism (CHI) is a significant disorder of hypoglycaemia caused by excessive and unregulated insulin secretion [1, 2]. CHI usually presents early in the neonatal period (Early-CHI), but later presentation (Late-CHI) is also well recognised [3–5]. A significant proportion of children with Congenital Hyperinsulinism (CHI) have adverse neurodevelopmental abnormalities in spite of improvements in medical care [3, 4, 6]. Identifying neurodevelopmental outcomes is a priority for children in follow up care; children with developmental needs may require additional support for physical and learning disabilities. The Vineland Adaptive Behavior Scales II© (VABS-II; Pearson Education Incorporated, San Antonio, Texas) parent report questionnaire is a tool that has been standardised for factors including gender, race, age and parental education, to identify children with developmental delay in the domains of communication, daily living skills, social skills, motor skills and behaviour. VABS-II is useful as an adaptive functioning inventory that could be completed at home without time consuming hospital visits and assessments. VABS-II has been used in a few children with CHI [7] but its reliability as a general screening tool to assess developmental delay in this population has not been assessed. VABS-II could be a credible tool to screen for adverse neurodevelopment in children with CHI, particularly at a younger age before formal time consuming cognitive testing is feasible. In this study, we have investigated the utility of VABS-II questionnaires as a parent report screening tool to identify developmental abnormalities in a relatively large population of children with CHI.

### Aims

We aimed to

1. investigate performance of VABS-II to identify developmental delay in CHI and
2. identify patient factors correlating with VABS-II scores.

## Methods

Parents of a cohort of children with CHI (*n* = 64), presenting consecutively between 2013 and 2015 to a specialist CHI treatment centre, completed the VABS-II questionnaire following consent. The diagnosis and treatment of CHI was based on established criteria and clinical practice [1, 2]. Medical and surgical treatment was individualised for each child. Patients’ characteristics and clinical outcome data were obtained from a database of patients. CHI was considered early (Early-CHI) if hypoglycaemia presentation was in the first month of life. Children who presented with hypoglycaemia later than one month had Late-CHI. In such children, neonatal records did not provide evidence for persistent hypoglycaemia. Following hospital discharge, children with Early and Late-CHI were assessed in the outpatient department by the clinical team comprising of clinicians, specialist nurse practitioners, dieticians, speech and language therapists and one clinical psychologist. VABS-II was discussed with parents as a routine screening tool for development after the age of 1 year.

VABS-II is a validated measure of intellectual and developmental functioning, and has been used in children with neonatal conditions [8], in neurological problems [9] and in children with genetic problems [10]. VABS-II has also been used in a small cohort of children with CHI, but its reliability as a screening tool has not been assessed [7]. Although VABS-II can be applied in children from birth, milder forms of developmental delay may not be apparent until an older age when clear progress in several developmental domains is obvious. Therefore, the minimum age for using the VABS-II was chosen at 18 months. No upper limit was specified; however as scores for motor skills in children > 6 years are estimates, analysis of VABS-II scores were run both with and without children > 6 years.

The VABS-II questionnaire was posted out to parents by the clinical psychologist (JN) who was trained and accredited to use and interpret the VABS-II. Where necessary she contacted parents by telephone to discuss queries about VABS-II responses. Populated questionnaires were returned to her for analysis in each of the domains of Communication, Daily Living Skills, Social Skills and Motor Skills [http://www.pearsonclinical.com/]. Domain scores were then compounded to derive the Adaptive Behaviour Composite (Total) score. For each VABS-II domain, scores were converted to Standard Deviation Scores (SDS) based on mean (SD) 15 (3). Total VABS-II scores were also converted to Total VABS-II SDS (VABS-II Total) based on mean (SD) 100 (15), (total scores are not additive). For each VABS-II domain and for composite scores, SDS < -2.0 was indicative of significant developmental delay. The Behaviour component of VABS-II was independently scored for internalising, externalising and total maladaptive behaviour scores, with raw scores having inverse correlation with behaviour outcomes. High scores corresponded to poor behaviour outcomes; for total Behaviour, scores ≥ 20 were considered unsatisfactory.

VABS-II was assessed for repeat variability by comparing initial report with a second report in a volunteer group (*n* = 7) after at least 1 year. The repeat assessment was performed to investigate if VABS-II demonstrated variability in relation to the timing of the test that could affect the interpretation of results. VABS-II was also analysed in a group of children with idiopathic ketotic hypoglycaemia (IKH) with normal neurodevelopment (*n* = 9) to assess test performance in an alternative condition of hypoglycaemia without significant adverse neurodevelopment.

VABS-II scores were compared with objective developmental assessment performed within 6 months of reporting. This assessment was performed by developmental paediatricians, clinical psychologists and educational psychologists who were unaware of VABS-II performance scores. The parents of children with CHI were unaware of the VABS-II scores and report until after the objective developmental assessment was performed. However, this testing was not centralised to the CHI centre; instead objective developmental assessment relied on methods specific to the local health authority, and were blinded to the results of VABS-II scores. However, derogation to local services meant that uniformity of formal testing was not maintained although the choice of formal developmental assessment allowed flexible testing in children of all abilities. The following developmental assessments were utilised - Wechsler Preschool and Primary Scale of Intelligence for Children – UK 4th Edition (WPPSI-IV), Wechsler Intelligence Scales for Children, 4th edition (WISC-IV UK), Movement Assessment Battery for Children – UK Second Edition (MAS-2), Bayley Scales of Infant and Toddler Development - Third Edition (Bayley-III) and Griffiths Developmental Scales. Objective assessment reports were available in 15 children, 6 from our centre and 9 from elsewhere. In the rest, information describing cognitive and developmental outcomes was obtained from patient clinical correspondence and reports obtained from community paediatricians and school assessments by educational psychologists. Formal or informal developmental testing was performed independent of VABS-II testing in all children in the cohort; therefore the study design did not control for the severity of neurodevelopmental outcome. As these tests varied in their reporting styles, no attempt was made to achieve uniformity of output, except for recording the presence or absence of delay in one or more domains of childhood development in the following categories – gross motor, fine motor, social and adaptive, communication and language. Brain neuroimaging was not performed routinely but reserved for clinical need.

VABS-II scores were also investigated for correlations with the timing of hypoglycaemia presentation, gender and phenotypes of CHI which included focal (solitary hyperfunctioning lesion in the pancreas), diffuse (hyperfunction in all islets in the pancreas) and transient (resolving hypoglycaemia, not requiring surgical treatment or long term medical therapy) forms, and treatment response. Transient and persistent CHI were defined as per previous descriptions [3, 11] with persistent forms indicating requirement for medication or need for pancreatic surgery. Genetic mutation status was determined by testing for known genes associated with CHI as previously described using standard methods [11]. Mutation status was positive if any pathologic mutation was present, in heterozygous or homozygous form, regardless of the mode of inheritance. CHI gene mutation was utilised as a proxy for greater severity, with known genetic forms having a greater requirement for medical or surgical therapy and less likely to achieve resolution of disease [11]. However, it is accepted that severity can be variable within individuals with the same genotype, between heterozygous, homozygous and compound heterozygous mutations and between K-ATP channel genes and non K-ATP channel genes. It is also possible that children without genetic mutations may have severe disease. While genetic mutation status is not an ideal severity marker, other markers such glucose infusion rates were not obtainable in patients with mild forms of CHI and those presenting late. We also utilised other severity markers such as response to diazoxide, transient or persistent CHI and requirement for surgery, although we accept that such markers are not validated, may be non-concordant, may represent a disproportionately severe end of the spectrum of disease and introduce bias in statistical correlations.

VABS-II was also tested in 9 children with IKH (age range 3.00 to 5.40 years), to assess performance in children with hypoglycaemia not due to CHI. These children presented with ketotic forms of hypoglycaemia in 2014-2015 and underwent investigations to exclude known causes of hypoglycaemia including CHI. Children who were recruited to the study did not have formal developmental assessment. However they were reviewed by the clinical psychologist who ensured normal neurodevelopment. Their hypoglycaemia was not treated by regular medication/food supplements but instead emergency hypoglycaemia prevention protocols incorporating additional carbohydrate intake during illness episodes were adopted.

Statistical analysis was performed by SPSS IBM© version 23.0 (IBM, New York, USA). VABS-II SDS between groups were compared by non-parametric tests. For repeat samples, paired tests with unequal variances were utilised. Probability of group membership was assessed by odds ratio, while sensitivity and specificity of tests were checked by receiver operating characteristic (ROC) curve analysis. The study was supported by the North West Research Ethics Committee, Project Reference Number 07/H1010/88.

## Results

**Fig. 1:**
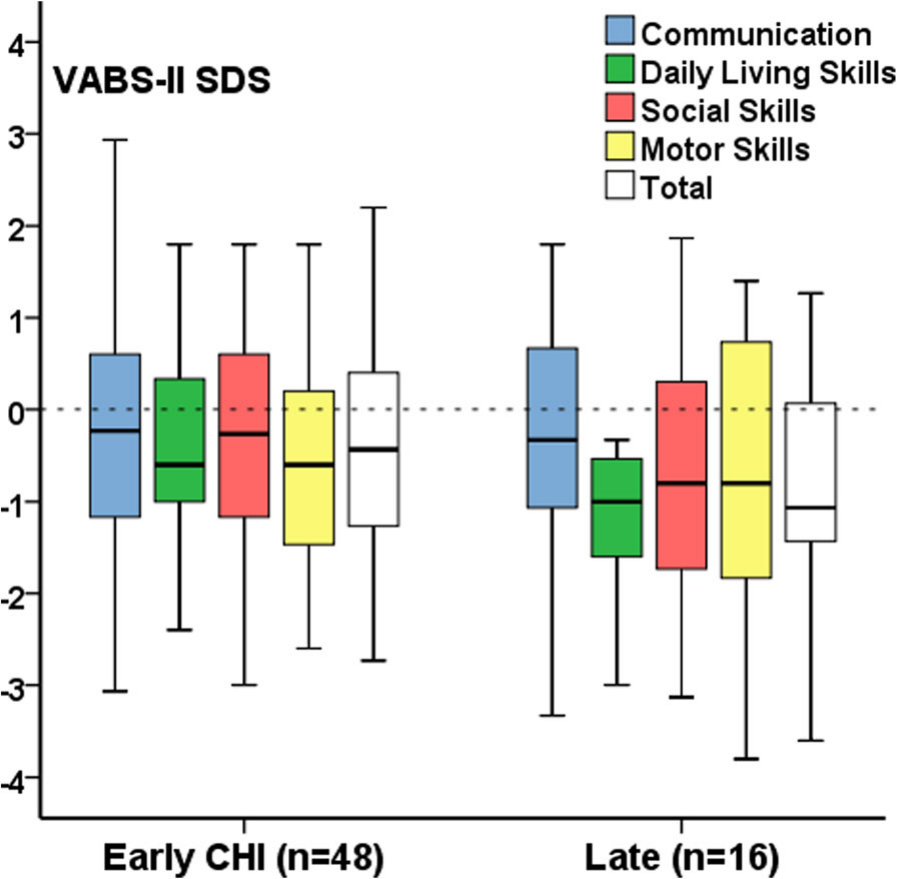
VABS-II scores have been expressed as SDS in patients with Early-CHI and Late-CHI for individual and adaptive behaviour composite (Total) domains. The reference line at 0 SDS represents the population mean with values < -2.0 representing significant deviation

VABS-II scores were completed in 64 (44 males, 69%) children with CHI at median (range) age 4.5 (1.5, 16.8) years, of whom 16 children (25%) were Late-CHI, with presentation at age 0.80 (0.30, 3.50) years. Individual and total VABS-II domain SDS scores (VABS-II scores) were below the expected population mean (range) [0 (-2.0, 2.0)] in the majority of patients (*n* = 41, 64%), with 9 (12%) being less than -2.0. VABS-II scores were as follows: Communication -0.26 (-3.33, 2.93), Daily Living Skills -0.73 (-3.60, 1.80), Social Skills -0.33 (-3.13, 1.87), Motor Skills -0.60 (-3.80, 1.80) and Total -0.48 (-3.60, 4.00). VABS-II scores were higher in Early-CHI than Late-CHI, although not reaching significance [-0.47 (-2.74, 4.00) v -0.70(-3.60, 1.27), *p* = 0.51] [Fig. 1]. VABS-II scores were in the normal population range [-0.33 (-1.73, 1.13)] for 9 children with IKH, in keeping with prior normal objective developmental assessment. VABS-II was repeated in 7 children (age range 3.00 to 9.30 years) with CHI; individual domain and total scores remained similar [paired samples test, *p* = 0.18 to *p* = 0.95], suggesting that the VABS-II was valid on repetition without significant deviation with advancing age.

### VABS-II scores in CHI correlate with developmental delay

VABS-II scores were correlated with developmental delay (affected in at least one developmental domain) identified by objective developmental assessment [Table 1]. Correlation was also sustained for involvement of one or more developmental domains regarded as a yes/no binary variable [odds ratio, OR (confidence interval, CI) 0.28 (0.13, 0.61), *p* = 0.001]. As motor skills scores were estimated in a group of children > 6 years old (*n* = 14), the analysis was re-run in the subgroup of children < 6 years old (*n* = 50) demonstrating a persistent strong correlation with developmental delay [OR (CI) 0.63 (0.44, 0.90), *p* = 0.012]. Lower VABS-II scores were also more likely in children with seizures at presentation [OR (CI) 0.49 (0.31, 0.76), *p* = 0.002] and epilepsy [OR (CI) 0.22 (0.09, 0.59), *p* = 0.003], further adding to the strength of association between VABS-II and developmental phenotyping in CHI.

**Table 1:** Individual and total VABS-II domain correlations with developmental delay by objective assessment performed by developmental paediatricians, clinical and educational psychologists

VABS-II domains correlating with developmental delay	Odds ratio	95% Confidence Intervals	*p* value
Total score	0.52	0.38; 0.73	<0.001
Communication	0.46	0.33; 0.65	<0.001
Daily Living Skills	0.63	0.44; 0.89	0.009
Social Skills	0.58	0.43; 0.78	<0.001
Motor Skills	0.54	0.38; 0.78	<0.001
Total Behaviour	1.21	1.04; 1.41	0.013
Externalising Behaviour	1.09	0.94; 1.27	0.251
Internalising Behaviour	1.30	1.09; 1.55	0.005

Development was delayed if one or more domains (gross motor, fine motor, social and adaptive, language and communication) were affected. Total and individual domain VABS-II scores showed higher probability of developmental delay with lower scores. Total Behaviour scores showed a higher probability for higher scores, mainly for internalising behaviour.

Receiver operating characteristic (ROC) curves [area under curve (AUC) (CI) 0.80 (0.68, 0.90), *p* < 0.001] were used to test sensitivity and specificity of VABS-II Total scores to identify developmental delay. At a VABS-II Total score -1.0 SDS, sensitivity was 63% and specificity was 79%. At a VABS-II Total score -2.0 SDS, sensitivity reduced to 26%, but specificity increased to 95%. Thus, while VABS-II Total scores < -2.0 SDS did not have a high pick up rate, an accurate diagnosis of developmental delay was ensured. For internalising behaviour scores, the ROC AUC associating with developmental delay was 0.78 (0.60, 0.95), *p* = 0.008 with a score of 15 showing sensitivity of 80% and specificity of 95%. With a behaviour score of 20, sensitivity reduced to 50%, but specificity increased to 91%. When VABS-II scores were analysed for predictive value for developmental delay in more than one domain [ROC AUC 0.80 (0.67, 0.92), *p* = 0.001], VABS-II Total score -1.8 SDS yielded sensitivity of 50% and specificity of 92%. Thus VABS-II and behaviour scores had high specificity for the diagnosis of developmental delay, with a low yield of undue false positive cases.

### VABS-II scores correlate with phenotypes of CHI

**Fig. 2:**
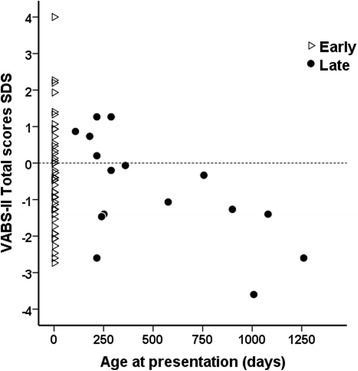
Scatterplot of VABS-II Total scores for age at presentation of hypoglycaemia with open triangles representing Early-CHI and filled circles representing Late-CHI. The reference line at 0 SDS indicates that most values in Late-CHI were below average. Older age at presentation was associated with lower VABS-II Total scores

**Fig. 3:**
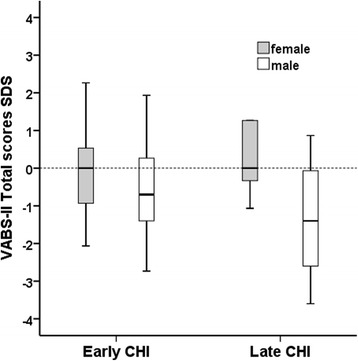
Clustered box and whisker plots of VABS-II Total scores in Early-CHI and Late-CHI for males (*white*) and females (*grey*) show adverse VABS-II scores in males presenting late

1. *Correlations with age and gender*:
  VABS-II Total scores were negatively correlated with advancing age at presentation of hypoglycaemia [adjusted R^2^ = 0.23, *p* = 0.018] [Fig. 2]. In linear regression (adjusted R^2^ = 0.19, *p* = 0.001), age at presentation (*p* = 0.024) and male gender (*p* = 0.036) were independently correlated with lower VABS-II scores. As a group, male scores were lower than females [-0.93 (-3.60, 4.0) v 0.00 (-2.07, 2.27), *p* = 0.020] regardless of their age. The difference was observed mainly within the domains of communication (*p* = 0.012) and social skills (*p* = 0.009), but not within daily living skills (*p* = 0.135) or motor skills (*p* = 0.187). Within Late-CHI, male scores were lower than females [-1.40 (-3.60,0.87) v +0.20 (-1.07,1.27), *p* = 0.014]. In analysis of covariance (R^2^ = 0.11, *p* = 0.011), male gender demonstrated an additional modest 6.5% effect (*p* = 0.04) on age at presentation to influence VABS-II Total scores. Therefore males presenting late carried higher risk for adverse neurodevelopmental outcomes [Fig. 3].

(2) Linking VABS-II scores to CHI severity:
  VABS-II scores were analysed for correlation with disease severity. Various phenotypic characteristics describing severity of CHI in our cohort has been provided in Appendix. Carriage of CHI gene mutations serving as a possible proxy for greater severity correlated with lower VABS-II Total scores in analysis of covariance with age at presentation as the covariate [adjusted R^2^ = 0.19, *p* = 0.039] with a modest effect size of 10%. In keeping with mutation status as a marker of severity, responsiveness to diazoxide was also positively correlated with VABS-II, i.e. unresponsiveness to diazoxide was associated with lower VABS-II Total scores (*p* = 0.019); however, no significant effects were noted for the following factors: transient or permanent CHI (*p* = 0.413), focal CHI (*p* = 0.742) and pancreatic surgery (*p* = 0.132).

### VABS-II Behaviour scores in CHI

**Fig. 4:**
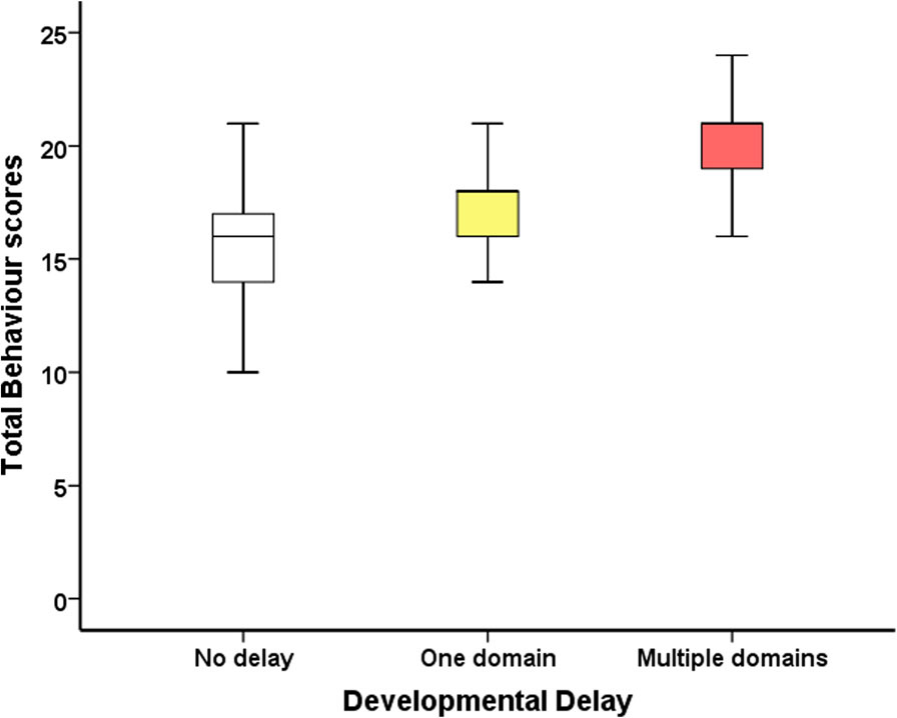
Box and whisker plot showing increasing total behaviour scores correlating with increasing severity of developmental delay

There were no differences in maladaptive behaviour scores between males and females for total, externalising and internalising behaviour scores (*p* = 0.949, *p* = 0.288, *p* = 0.710 respectively). Similarly, no differences in Early-CHI and Late-CHI were observed in total and each behaviour domain (*p* = 0.226, *p* = 0.760, *p* = 0.096). When adjusting for gender, age at presentation did not correlate with behaviour (*p* = 0.811, *p* = 0.744, *p* = 0.561). However, greater total behaviour scores were associated with developmental delay [OR (95%CI) 1.21 (1.04, 1.41), *p* = 0.013] [Fig. 4], more so for internalising behaviour [OR (95%CI) 1.30 (1.09, 1.55), *p* = 0.005] than for externalising behaviour (*p* = 0.251). However, the combination of total Vineland scores and total maladaptive behaviour scores (as a composite measure) to predict developmental delay was not additionally informative (*p* = 0.836). Therefore it follows that total maladaptive behaviour scores or total VABS-II scores are individually correlated with developmental delay but without additive effects.

## Discussion

In this study, we have assessed the reliability of VABS-II as a screening tool for developmental delay in children with CHI. VABS-II and maladaptive behaviour scores individually correlated with developmental delay. VABS-II had high specificity, indicating accuracy of screening investigations for formal developmental assessments. Male gender and late age of hypoglycaemia presentation were risk factors for lower VABS-II scores. Diazoxide unresponsiveness and carriage of CHI genetic mutations, proxy markers of severity of CHI, were also associated with lower VABS-II scores.

Although VABS-II is applied widely to children with different conditions, its utility and accuracy in children with CHI has not been investigated previously. Our study has reviewed the performance of VABS-II in a relatively large cohort of children with the rare disease of CHI, over a two year period and correlated parent reports with objective clinical developmental examination. Further, our study examined VABS-II in IKH, where hypoglycaemia is not usually associated with significant brain injury; as expected VABS-II showed normal variation in IKH individuals. Our study also found consistency in repeating VABS-II in later life, thereby eliminating age dependent bias. The strength of association of VABS-II with developmental delay and the observation that a third of children have abnormal neurodevelopment [3] makes a compelling case to use VABS-II routinely in children with CHI with any clinical concern about neurodevelopment.

Although VABS-II demonstrated strong correlation with developmental delay and had high specificity, formal assessment methods to diagnose developmental delay were not uniform. However as cognitive testing using inventories such as WISC-IV UK can be time consuming, require trained psychologists and may be unsuitable for younger children, flexibility of objective developmental assessment was inevitable. Nonetheless, the non-uniformity of formal developmental testing remains a weakness, which may account for the skewed sensitivity values of VABS-II. It does reflect the variability that exists within clinical practices, however.

The study design did not specify formal developmental testing for all patients. It is possible that more patients who had severe neurodevelopmental delay were tested by formal methods than those with mild delay. However, in those tested formally in our cohort, nearly half were tested routinely. In such patients, testing was not biased by the severity of adverse neurodevelopment. It is possible that the sensitivity of VABS-II for milder adverse neurodevelopment is higher than that observed. An important corollary from our observations is the need for a more rigorous test of performance of VABS-II in the detection of milder forms of developmental delay. We recognise that mild abnormalities due to early life hypoglycaemia may not be reversed by early detection by VABS-II; however it is possible that early therapeutic interventions and adaptions in home and learning environments could be beneficial. The identification of relatively subtle neurodevelopmental abnormalities and interventions on quality of life could be a logical follow on study.

In this study, brain imaging was not performed as routine. Instead brain magnetic resonance imaging was prioritised for clinical need as in a previous observational study [3]. While the absence of brain imaging could be construed as a weakness in the study design, the value of routine brain imaging, a resource intense investigation, has not been substantiated for neurodevelopmental screening of CHI other than to identify the topography of lesions [12].

VABS-II had high specificity but low sensitivity for developmental delay. Therefore in the context of screening, VABS-II may not be relied upon as a primary tool for developmental referral in CHI. However, in clinical practice, developmental concerns are routinely discussed in outpatient follow up, which would obviate the requirement for VABS-II to be a highly sensitive instrument. The clinical suspicion of delayed development could be construed as a first line screening test, followed by VABS-II as a next step screening tool. The high specificity of VABS-II in the context of clinical concerns should generate sufficient concern to trigger referral for formal developmental assessments.

It is not clear why males with CHI have lower VABS-II scores than females. Frequency of males was greater in our cohort; however, gender as a variable was controlled in analysis at several levels. Our study has raised the interesting question whether gender difference could be an independent predictor over hypoglycaemic brain injury to cause intellectual disability. This question cannot be answered within the remit of our study design and needs to be examined in larger cohorts. Recent observations suggest modulation of sensitivity of arcuate nuclei to hypoglycaemia by suprachiasmatic nuclei in male rats [13]; it remains to be seen if similar mechanisms apply to male brains in children with CHI.

Our study shows that children presenting later with CHI have lower VABS-II scores, correlating with adverse developmental outcomes. This observation remains even after adjustment for male gender, another risk factor for lower VABS-II scores. CHI usually presents in the neonatal period; it is possible that patients with Late CHI had neonatal hypoglycaemia but that the diagnosis was achieved later. However, examination of neonatal records makes this less likely, although not impossible. A previous observation of later presentation has been associated with long term neurological disabilities [4] suggesting recurrent and unrecognised hypoglycaemia impacting on developmental outcomes. In our cohort, it is likely that recurrent hypoglycaemia was missed prior to the eventual diagnosis of CHI. Such hypoglycaemic episodes could be responsible for greater severity of adverse neurodevelopment in those with Late CHI. The inverse correlation with age at presentation indicates the need for early recognition and treatment of hypoglycaemia in early life [1, 14]. As expected, diazoxide unresponsiveness and carriage of genetic mutations, proxy markers of severity of CHI, were associated with lower VABS-II scores. It therefore follows that treatment response and gene mutation testing in the initial phase of clinical management may provide prognostic markers to determine neurodevelopmental trajectories in CHI. It is well recognised that patients undergoing pancreatectomy for CHI have a high frequency of neurobehavioural deficits [15]. Our study adds further evidence to the impact of severity phenotyping on long-term outcomes.

## Conclusions

We have evaluated the performance of Vineland Adaptive Behavior Scales, 2nd edition (VABS-II) in children with CHI and noted lower scores correlating with the presence of developmental delay with high specificity. Male gender, late age at presentation and severity of CHI are risk factors for adverse outcomes. VABS-II can be reliably used in neurodevelopmental follow up of CHI patients to trigger formal developmental assessment.

## Appendix

**Table 2:** Clinical characteristics associated with severity of CHI

	Late or Early Presenting	Mutation	Transient or Persistent	Responsive to Medication	Surgery	Focal or Diffuse	Total VABS II SDS
# 1	E	Paternal ABCC8	P	Unresponsive	SP	D	-0.9
# 2	L	Negative	P	Diazoxide	No	D	-2.6
# 3	L	Negative	P	Diazoxide	No	D	-1.3
# 4	E	Compound heterozygous ABCC8	P	Unresponsive	SP	D	-0.9
# 5	L	Negative	P	Diazoxide	No	D	-1.1
# 6	L	GCK	P	Diazoxide	No	D	-2.6
# 7	E	Negative	T	Diazoxide	No	D	-1.9
# 8	E	Paternal KCNJ11	T	Diazoxide	No	D	-1.1
# 9	E	Paternal ABCC8	P	Unresponsive	FL	F	-2.3
# 10	E	Negative	P	Diazoxide	No	D	-2.5
# 11	E	Negative	P	Diazoxide	No	D	-1.3
# 12	L	Negative	P	Unresponsive	SP	D	-3.6
# 13	E	Homozygous ABCC8	P	Unresponsive	SP	D	-1.1
# 14	L	Negative	P	Diazoxide	No	D	0.7
# 15	E	Negative	T	Diazoxide	No	D	1.9
# 16	E	Negative	T	Diazoxide	No	D	-0.8
# 17	E	Negative	P	Unresponsive	SP	D	1.1
# 18	E	Maternal KCNJ11	T	Diazoxide	No	D	-0.4
# 19	L	Negative	T	Diazoxide	No	D	0.2
# 20	E	Negative	T	Diazoxide	No	D	-0.5
# 21	E	Maternal ABCC8	T	Diazoxide	No	D	0.1
# 22	E	Paternal ABCC8	T	Diazoxide	No	D	-2.1
# 23	E	Maternal ABCC8	P	Diazoxide	No	D	-1.7
# 24	L	Negative	P	Diazoxide	No	D	-1.4
# 25	E	Negative	T	Glucose	No	D	0.3
# 26	L	Negative	P	Diazoxide	No	D	-0.3
# 27	E	Negative	T	Diazoxide	No	D	-1.5
# 28	E	de novo ABCC8	P	Diazoxide	No	D	2.3
# 29	E	Negative	T	Diazoxide	No	D	-0.8
# 30	L	Negative	P	Diazoxide	No	D	-0.2
# 31	E	Negative	T	Glucose	No	D	-2.1
# 32	L	Negative	T	Diazoxide	No	D	-0.1
# 33	L	Paternal ABCC8	T	Octreotide	No	D	-1.4
# 34	E	Paternal ABCC8	T	Octreotide	No	D	-2.7
# 35	L	Negative	T	Diazoxide	No	D	1.3
# 36	E	Maternal ABCC8	T	Diazoxide	No	D	0.0
# 37	L	Paternal ABCC8	P	Diazoxide	FL	F	0.9
# 38	E	Negative	T	Diazoxide	No	D	0.7
# 39	E	Negative	T	Glucose	No	D	1.4
# 40	E	Negative	T	Diazoxide	No	D	-1.3
# 41	E	Negative	P	Diazoxide	No	D	-1.3
# 42	E	Negative	T	Diazoxide	No	D	-1.4
# 43	E	Negative	T	Diazoxide	No	D	-1.3
# 44	E	Negative	P	Diazoxide	No	D	1.3
# 45	E	Negative	T	Diazoxide	No	D	4.0
# 46	L	Negative	T	Diazoxide	No	D	-1.5
# 47	L	Negative	T	Diazoxide	No	D	1.3
# 48	E	Negative	T	Diazoxide	No	D	0.5
# 49	E	Paternal KCNJ11	T	Octreotide	No	D	-0.3
# 50	E	Negative	T	Diazoxide	No	D	-0.2
# 51	E	Negative	P	Diazoxide	No	D	0.0
# 52	E	Negative	P	Diazoxide	No	D	-0.9
# 53	E	Negative	T	Diazoxide	No	D	0.3
# 54	E	Homozygous ABCC8	P	Unresponsive	SP	D	-2.6
# 55	E	Negative	T	Diazoxide	No	D	0.9
# 56	E	Homozygous ABCC8	P	Octreotide	No	D	-0.4
# 57	E	Negative	T	Diazoxide	No	D	-0.6
# 58	E	Negative	P	Diazoxide	No	D	2.2
# 59	E	Paternal KCNJ11	P	Octreotide	No	D	-1.9
# 60	E	Negative	T	Diazoxide	No	D	-0.4
# 61	E	Compound heterozygous ABCC8	P	Octreotide	No	D	0.9
# 62	E	Negative	T	Diazoxide	No	D	0.5
# 63	E	Negative	T	Diazoxide	No	D	-0.5
# 64	E	Negative	T	Diazoxide	No	D	0.1

Table of clinical descriptors in patients with CHI with time of presentation, genetic status, resolution of hypoglycaemia, response to medication, requirement for pancreatic surgery and Total VABS-II scores

*E* early, *L* late, *T* transient, *P* permanent, *D* diffuse, *F* focal, *FL* focal lesionectomy, *SP* subtotal pancreatectomy

All patients on octreotide had previously failed to respond to diazoxide

Outcomes of surgery: Patients with focal CHI were cured after focal lesionectomy. Patient # 1 and # 13 became diabetic post-pancreatectomy; patient # 12 and # 54 are euglycaemic post-pancreatectomy; patients # 4 and #17 have impaired glucose tolerance

## Abbreviations

AUC: Area under the curve
CHI: Congenital hyperinsulinism
CI: Confidence intervals
IKH: Idiopathic ketotic hypoglycaemia
MAS-2: Movement assessment battery for children – UK Second Edition
OR: Odds ratio
ROC: Receiver operating characteristic
SDS: Standard deviation scores
VABS-II: Vineland adaptive behavior scales, second edition
WISC-IV UK: Wechsler intelligence scales for children, 4th edition
WPPSI-IV: Wechsler preschool and primary scale of intelligence for children – UK 4th Edition

## References

[1] Arnoux JB, Verkarre V, Saint-Martin C, Montravers F, Brassier A, Valayannopoulos V, Brunelle F, Fournet JC, Robert JJ, Aigrain Y (2012). Congenital hyperinsulinism: current trends in diagnosis and therapy. Orphanet J Rare Dis.

[2] Banerjee I, Avatapalle B, Padidela R, Stevens A, Cosgrove K, Clayton P, Dunne M (2013). Integrating genetic and imaging investigations into the clinical management of congenital hyperinsulinism. Clin Endocrinol (Oxf).

[3] Avatapalle HB, Banerjee I, Shah S, Pryce M, Nicholson J, Rigby L, Caine L, Didi M, Skae M, Ehtisham S (2013). Abnormal neurodevelopmental outcomes are common in children with transient congenital hyperinsulinism. Front Endocrinol (Lausanne).

[4] Meissner T, Wendel U, Burgard P, Schaetzle S, Mayatepek E (2003). Long-term follow-up of 114 patients with congenital hyperinsulinism. Eur J Endocrinol.

[5] Touati G, Poggi-Travert F, Ogier De Baulny H, Rahier J, Brunelle F, Nihoul-Fekete C (1998). Long-term treatment of persistent hyperinsulinaemic hypoglycaemia of infancy with diazoxide: a retrospective review of 77 cases and analysis of efficacy-predicting criteria. Eur J Pediatr.

[6] Menni F, de Lonlay P, Sevin C, Touati G, Peigne C, Barbier V, Nihoul-Fekete C, Saudubray JM, Robert JJ (2001). Neurologic outcomes of 90 neonates and infants with persistent hyperinsulinemic hypoglycemia. Pediatrics.

[7] Levy-Shraga Y, Pinhas-Hamiel O, Kraus-Houminer E, Landau H, Mazor-Aronovitch K, Modan-Moses D, Gillis D, Koren I, Dollberg D, Gabis LV (2013). Cognitive and developmental outcome of conservatively treated children with congenital hyperinsulinism. J Pediatr Endocrinol Metab.

[8] Hack M (1999). Consideration of the use of health status, functional outcome, and quality-of-life to monitor neonatal intensive care practice. Pediatrics.

[9] Berg AT, Caplan R, Baca CB, Vickrey BG (2013). Adaptive behavior and later school achievement in children with early-onset epilepsy. Dev Med Child Neurol.

[10] Msall ME, Tremont MR (1999). Measuring functional status in children with genetic impairments. Am J Med Genet.

[11] Banerjee I, Skae M, Flanagan SE, Rigby L, Patel L, Didi M, Blair J, Ehtisham S, Ellard S, Cosgrove KE (2011). The contribution of rapid KATP channel gene mutation analysis to the clinical management of children with congenital hyperinsulinism. Eur J Endocrinol.

[12] Gataullina S, Lonlay PD, Dellatolas G, Valayannapoulos V, Napuri S, Damaj L, Touati G, Altuzarra C, Dulac O, Boddaert N (2013). Topography of brain damage in metabolic hypoglycaemia is determined by age at which hypoglycaemia occurred. Dev Med Child Neurol.

[13] Herrera-Moro Chao D, Leon-Mercado L, Foppen E, Guzman-Ruiz M, Basualdo MC, Escobar C, Buijs RM (2016). The Suprachiasmatic nucleus modulates the sensitivity of Arcuate nucleus to hypoglycemia in the male rat. Endocrinology.

[14] De Leon DD, Stanley CA (2007). Mechanisms of Disease: advances in diagnosis and treatment of hyperinsulinism in neonates. Nat Clin Pract Endocrinol Metab.

[15] Lord K, Radcliffe J, Gallagher PR, Adzick NS, Stanley CA, De Leon DD (2015). High risk of diabetes and neurobehavioral deficits in individuals with surgically treated hyperinsulinism. J Clin Endocrinol Metab.

